# Recurrent Systemic Embolization From Bicuspid Aortic Valve Endocarditis in the Setting of Anti-coagulation Use

**DOI:** 10.7759/cureus.73187

**Published:** 2024-11-07

**Authors:** Soomal Rafique, Salvador Fernandez, Saliha Saleem, Sami Akram

**Affiliations:** 1 Internal Medicine, Southern Illinois University School of Medicine, Springfield, USA; 2 Infectious Diseases, Southern Illinois University School of Medicine, Springfield, USA

**Keywords:** antibiotics therapy, bicuspid aortic valve disease, infective endocarditis, intraoperative/postoperative anticoagulation, septic emboli, surgical replacement of valve

## Abstract

Infective endocarditis (IE) is a systemic disease with a high mortality rate even with intravenous antibiotic therapy. Abnormal valves, including bicuspid aortic valves (BAV), are particularly prone to it compared to normal valves. We present a 22-year-old female who was initially admitted for the management of acute splenic infarction when she was diagnosed with a bicuspid aortic valve. With no evidence of a cardiac source of the embolus, she was discharged on anti-coagulation. However, she returned with acute toe ischemia in a few days. She was found to have *Streptococcus mitis* bacteremia, multiple sub-centimeter aortic valve vegetations on trans-esophageal echocardiogram (TEE), and was subsequently diagnosed with IE. After 3 weeks of IV antibiotics, she presented with thalamic stroke. Our case underscores the challenges in managing IE, particularly in young patients with BAV. Early recognition and aggressive treatment, regardless of vegetation size, and avoidance of anti-coagulation are crucial to mitigate embolic complications.

## Introduction

The bicuspid aortic valve, whether familial or sporadic, is found in 0.5-2% of the population. Being the most common congenital heart anomaly, it is also associated with aortic root dilatation [1]. The bicuspid aortic valve is associated with several conditions including, but not limited to, coarctation of the aorta and certain syndromes like Turner's, William's, and Shone syndrome [2]. While it usually remains asymptomatic during childhood and is often diagnosed incidentally, adults typically receive a diagnosis due to complications, including valvular dysfunction, aortic aneurysm, or aortic dissection [3]. Recent studies suggest a 2-5% risk of infective endocarditis in these patients, and about 80% of significant vegetation on the left side can lead to embolization of the spleen and brain [4]. We present a case involving sub-centimeter, ambiguous vegetations on the aortic valve, which have resulted in repeated episodes of septic emboli.

## Case presentation

A 22-year-old female with a history of gastroesophageal reflux disease (GERD), bipolar 2 disorder, borderline personality disorder, post-traumatic stress disorder (PTSD), and cannabis use disorder presented to the emergency room with sudden-onset epigastric pain radiating to the back for one day. She was on lamotrigine and mentioned inconsistent use of oral contraceptives that were prescribed three months ago, although she denied any history of sick contacts, traveling, or changes in her medications.

Vitals and physical examination

On presentation, her blood pressure was 122/80 mmHg, her heart rate was 87 beats per minute, and she saturated 99% on room air. Abdominal examination revealed mild left upper quadrant tenderness. The rest of the examination was unremarkable. While in the emergency department, she received intravenous fluids, anti-emetics, and morphine.

Investigation/Work-up

**Figure 1:**
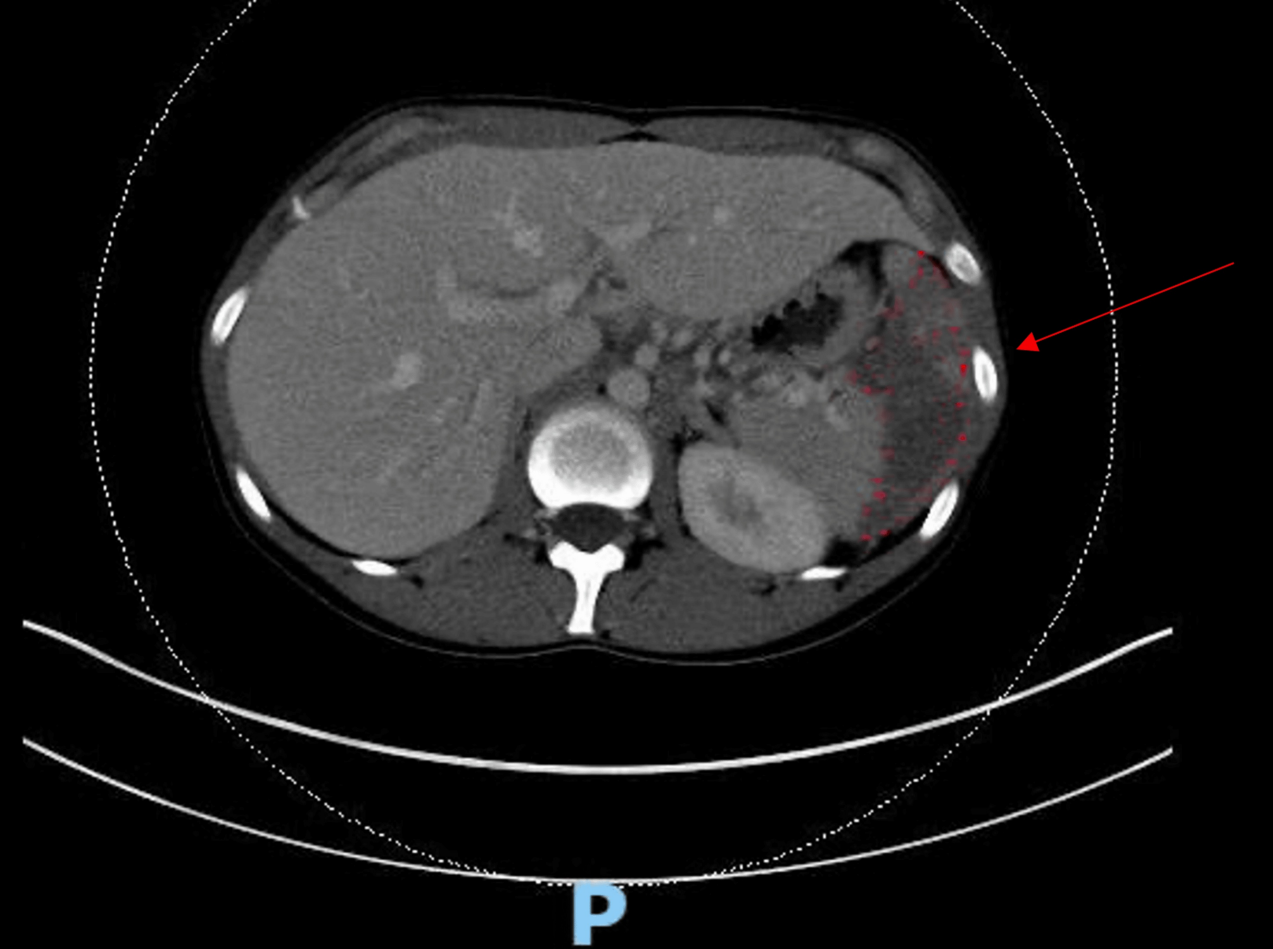
Large acute infarct in the mid to lower portion of the spleen

Initial labs were notable for normocytic anemia (hemoglobin 11.7 g/dl) and neutrophilic leukocytosis (WBC count 12.8 K/cumm). CT of the abdomen revealed a relatively large acute splenic infarct without splenomegaly and minimal dependent left lower lobe atelectasis of the lung (Figure 1).

Clinical course

Given the CT findings mentioned above, a broad differential was considered. D-dimers, fibrinogen, and factor VIII levels were elevated, with slightly low protein C levels (Table 1). Workup for sickle cell anemia, infectious mononucleosis, protein S anti-thrombin III, Factor V Leiden mutation, myeloproliferative disorder, lymphoma, hepatitis B and HIV infections, and lupus was negative.

**Table 1:** Pertinent initial work-up INR: International Normalized Ratio; APTT: Activated partial thromboplastin time

Pertinent laboratory work-up		
Parameter	Result	Normal Range
Hemoglobin (g/dl)	11.7	12-16
White cell count (x10^3^/ul)	12.8	4-11
Platelets (x 10^3^/ul)	402	130-400
Prothrombin time (sec)	14.5	11.6-14.5
INR	1.1	0.9-1.1
APTT (sec)	25.4	22.9-35.1
D-dimers (mcg/ml)	1.41	<0.5
Fibrinogen (mg/dl)	591	200-400
Protein C (%)	75	80-150
Protein S (% inhibition)	74	60-150
Factor VIII (%)	238	50-150
Antithrombin III (%)	96	80-120
Hemoglobin Electrophoresis (HgA %)	96.6	95.0-97.9

**Figure 2:**
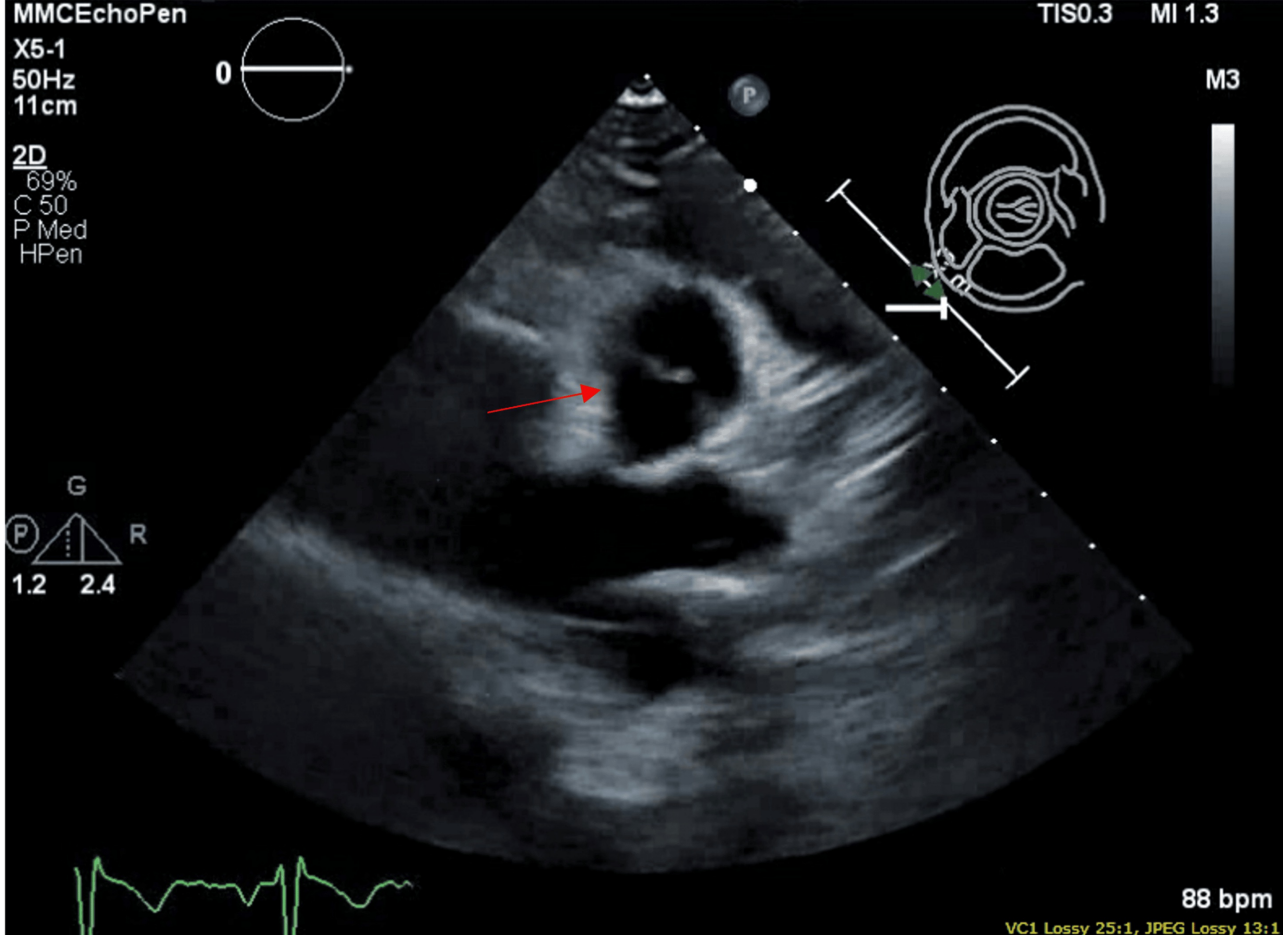
TTE showing bicuspid aortic valve with no evidence of vegetation TTE: transthoracic echocardiogram

A transthoracic echocardiogram (TTE) was ordered to rule out the cardiac source of embolus, which revealed a bicuspid aortic valve (Figure 2) with normal left ventricular ejection fraction, right ventricular function, and rest of the valves. Blood cultures were negative. General surgery was consulted, and no acute surgical intervention was suggested. A hematology-oncology consultation was made that recommended 72 hours of intravenous heparin, followed by apixaban on discharge.

**Figure 3:**
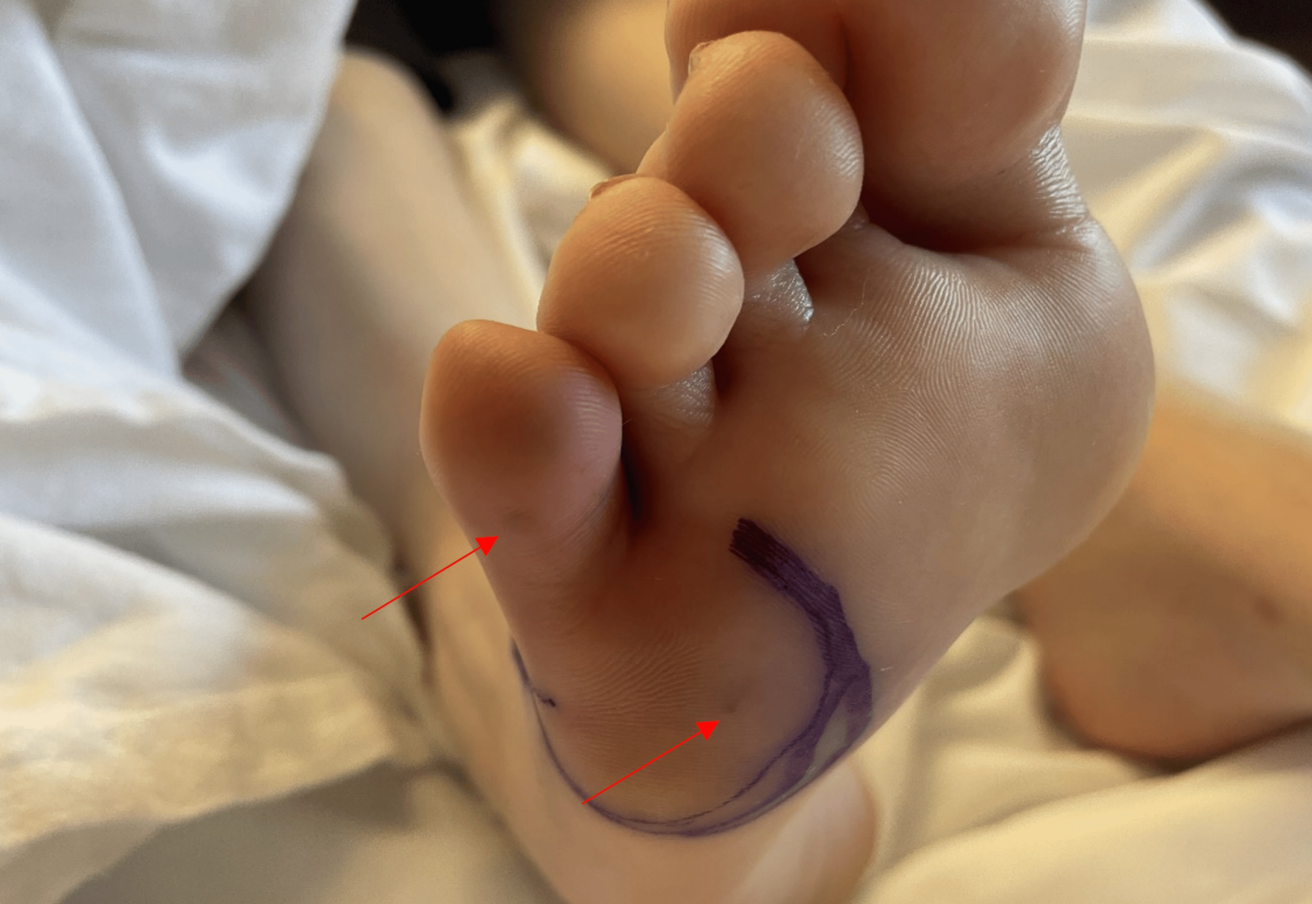
Foot examination revealing Osler's nodes on the ventral surface of the lateral foot and 5th digit.

While on the starter pack of apixaban, the patient returned to the emergency room with sudden onset toe pain in the right foot seven days after the previous discharge. She now reported fevers and chills intermittently for the past one month. On arrival, she had a fever of 38^o^C but otherwise remained hemodynamically stable. Physical examination of the right foot revealed erythematous and edematous 5th digit, with warmth and tenderness to touch and decreased range of motion of the digit. There were some tender brown lesions on the ventral surface of the lateral foot, likely Osler nodes (Figure 3).

**Figure 4:**
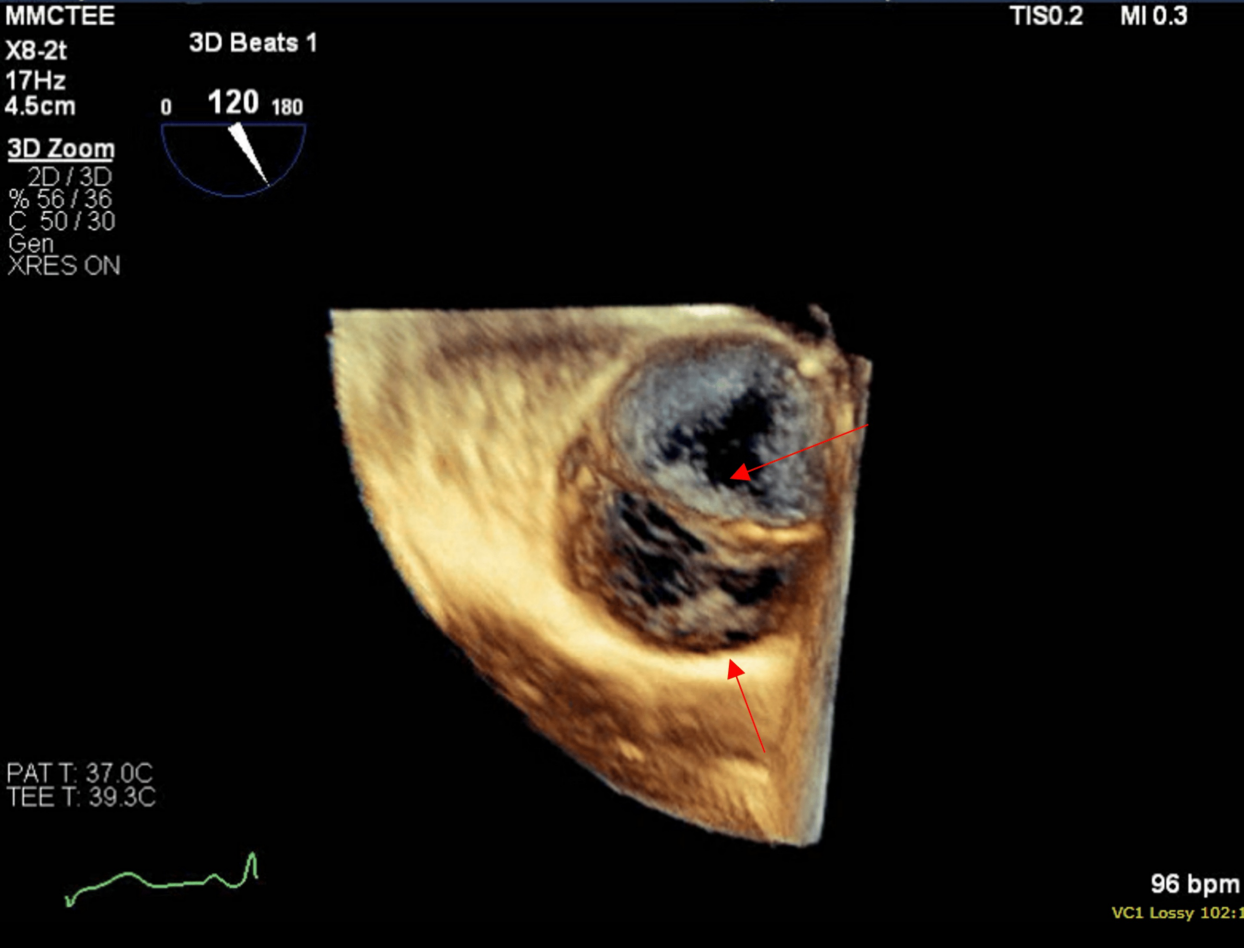
Transesophageal echocardiogram revealing bicuspid aortic valve with valvular vegetation

She had leukocytosis of 13.1 k/cumm and elevated CRP of 17.4 ng/l. X-ray of the foot was normal, and CT angiography of the aorta with extremity runoff showed normal arteries and known splenic infarct without abscess. Two sets of blood cultures were positive for gram-positive cocci, which later finalized as Streptococcus mitis. Suspicion for septic embolus was made, anti-coagulation was discontinued, and Infectious Diseases commenced antibiotics as the patient met one major (positive blood cultures) and 3 minor criteria (predisposing heart condition, fever, and vascular phenomenon) for infective endocarditis. Trans-esophageal echocardiogram (TEE) revealed a bicuspid aortic valve with mild aortic insufficiency, an echo density on the anterior cusp that measured 0.5 x 0.4cm, another lesion measuring 0.6 x 0.2 cm on the aortic valve annulus (Figure 4). Cardiothoracic surgery was consulted, medical management with IV antibiotics was recommended with follow-up TEE after six weeks, and the patient was discharged on IV penicillin G for six weeks after negative blood cultures. A dental source with jaw panorex was ruled out.

**Figure 5:**
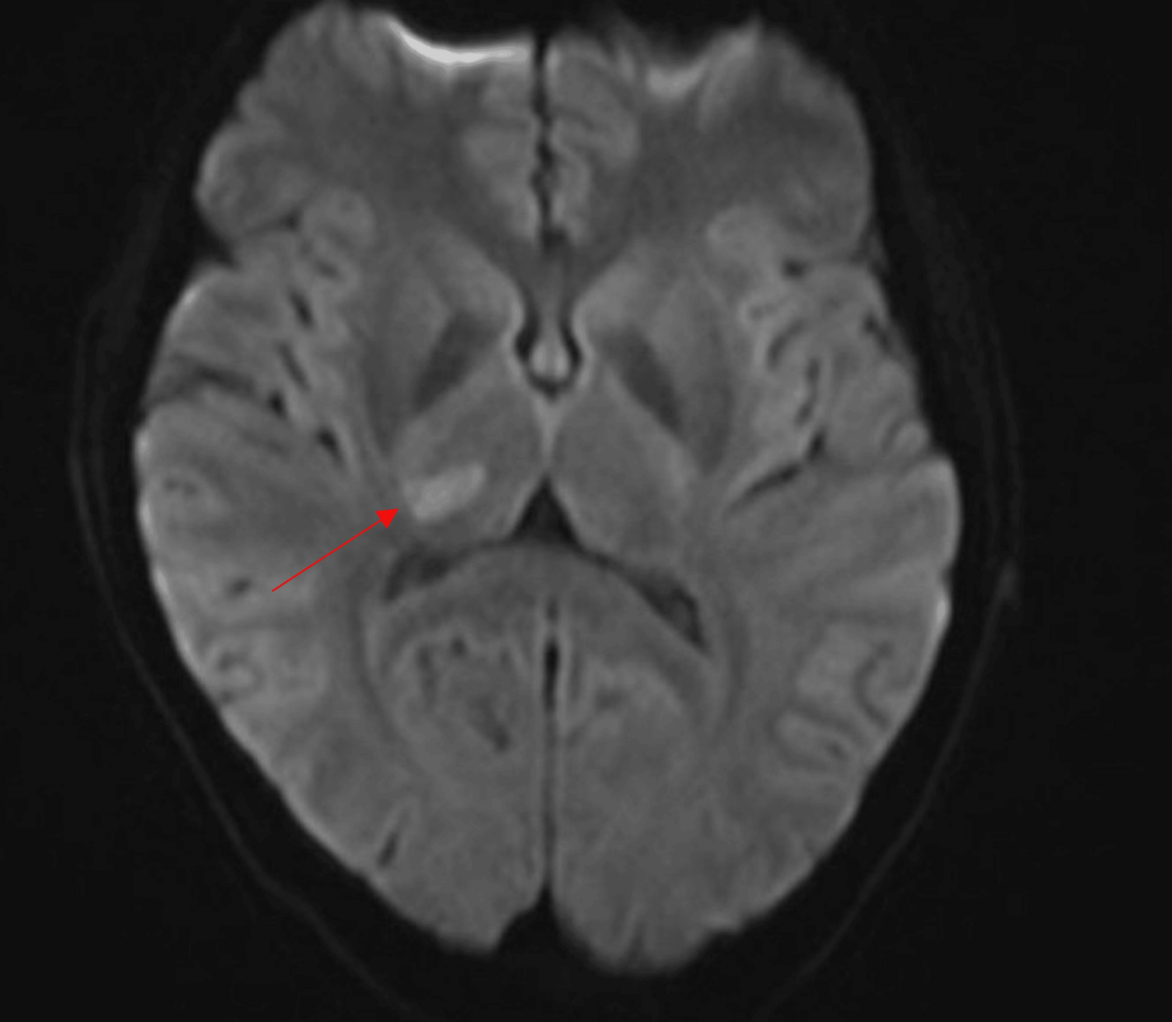
MRI brain shows right thalamic infarct

Three weeks later, the patient returned with a severe headache different from her usual migraines, blurred vision, and generalized body weakness. Examination revealed decreased sensation to light touch in the left trigeminal V1, V2, and V3 territories and left upper and lower extremities. Labs on presentation were mostly unremarkable. A magnetic resonance image of the brain revealed an acute infarction of the right thalamus measuring 13 x 7 mm without hemorrhagic conversion (Figure 5).

**Figure 6:**
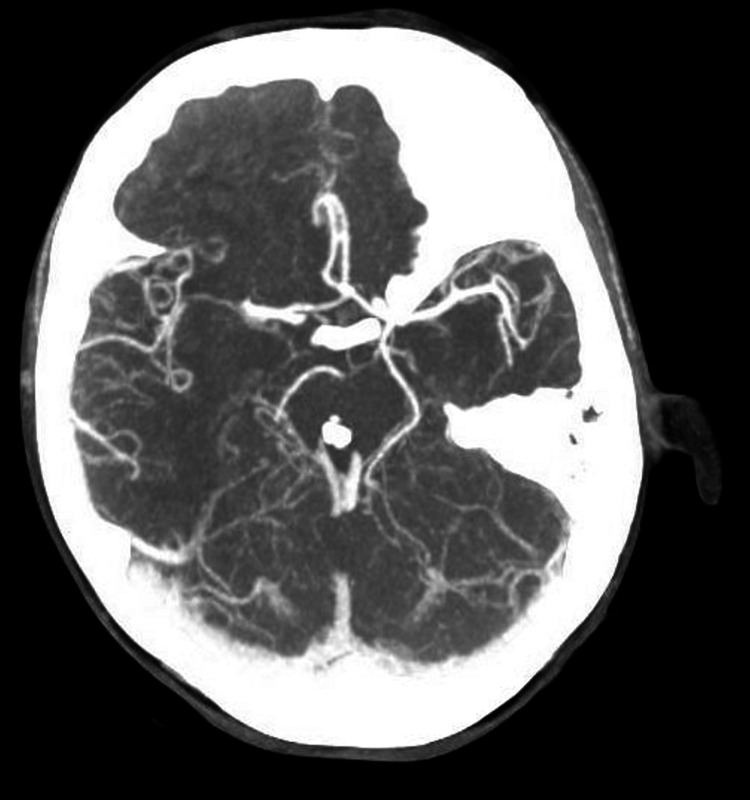
CT angiography of the head showing no significant arterial stenosis, arterial occlusion, intracranial aneurysm, or AVMs AVM: Arteriovenous malformations

CT angiography of the head and neck showed stable size of acute non-hemorrhagic infarct at the right thalamus. However, no significant arterial stenosis, arterial occlusion, intracranial aneurysm, or arteriovenous malformations (AVMs) were appreciated (Figure 6). Glycated hemoglobin and lipid panel were within normal range. The stroke team did not recommend any further work-up or anti-coagulation as the source was thought to be the embolism from aortic valve endocarditis.

**Figure 7:**
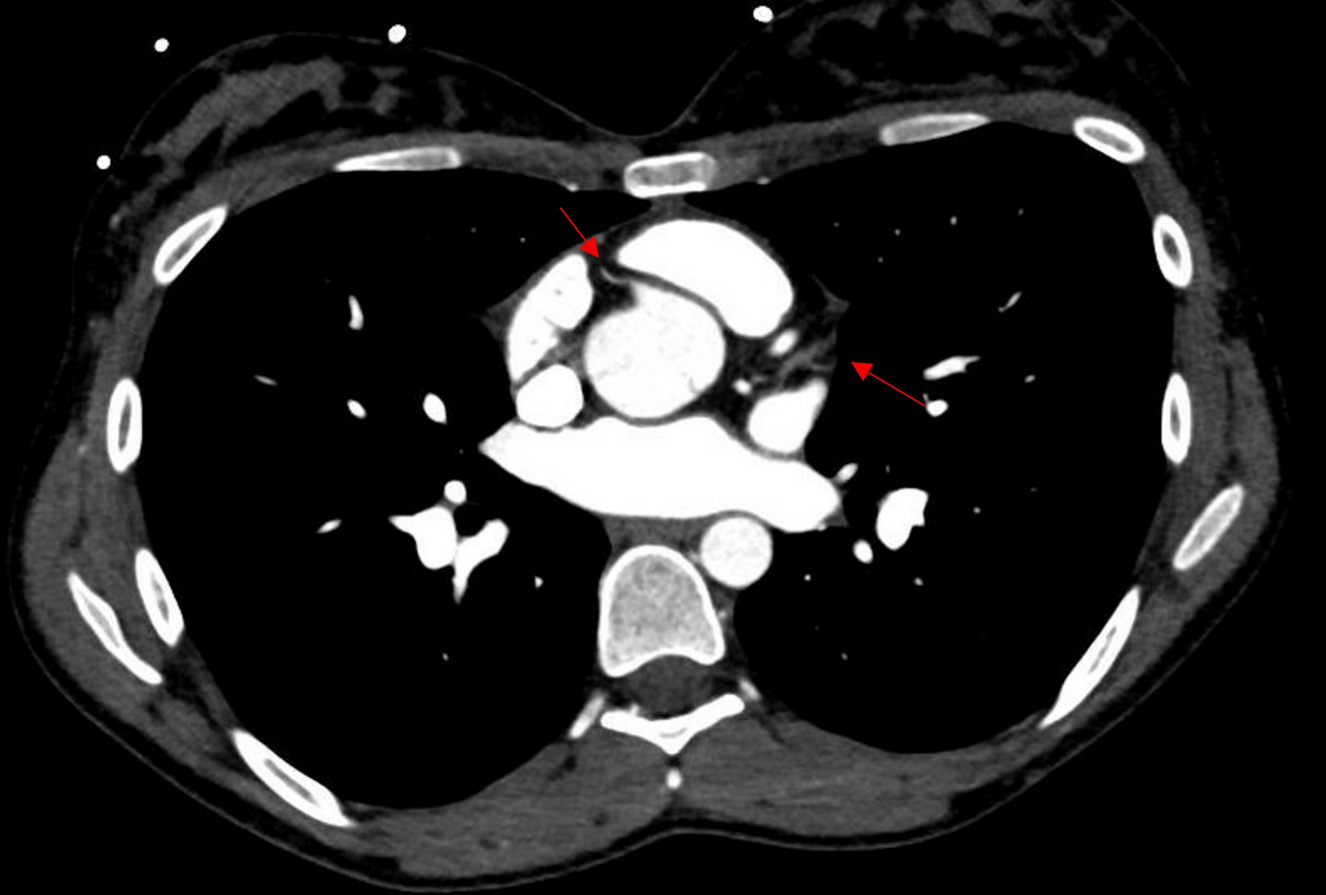
CT angiogram of the coronaries showing patent coronary arteries

Cardiothoracic surgery re-evaluated the patient for the need for surgery, with repeat TEE showing healing vegetations at the aortic valve cusps with a decrease in size and mild aortic regurgitation that was worse than before. A coronary CT scan was also performed, as the left heart coronary angiogram was contraindicated, and no abnormalities of coronary vessels were observed (Figure 7). No acute surgical intervention was planned, and the patient was discharged on statin therapy with outpatient surgical follow-up. There are currently plans for mechanical aortic valve replacement per cardiothoracic surgery with a preceding diagnostic cerebral angiogram to rule out mycotic aneurysms.

## Discussion

Bicuspid aortic valve (BAV) is the most common congenital heart defect, with a 2:1 male-to-female ratio [5]. While the specific genetic cause remains largely unidentified, it is typically inherited as an autosomal dominant trait, with approximately 10% of screened siblings also affected [6]. BAV is classified into three common morphologies based on cusp fusion and the presence of a raphe. The most prevalent type, accounting for 70-80% of cases, is type 1, characterized by fusion of the right and left coronary cusps, known as true BAV [7]. Diagnosis is usually made via TTE, which has a sensitivity of 70-92% and specificity of 96%. However, in cases where obtaining clear images is challenging or for assessing aortopathy or vegetation, TEE, cardiac CT, or cardiac magnetic resonance imaging (CMR) can be beneficial [8].

Patients with BAV can experience complications at any stage, leading to a condition known as valvuloaortopathy. This includes risks of aortic stenosis, regurgitation, and infective endocarditis, as well as an increased likelihood of surgical intervention and perivalvular abscesses. Notably, about three-quarters of patients with native valve infective endocarditis have underlying heart conditions, including BAV. Despite these potential complications, the 25-year survival rate post-diagnosis is comparable to that of the general population [9].

Infective endocarditis (IE) has a higher incidence in males and affects about 2% of the BAV population. BAV-associated IE carries an increased risk of abscess formation, but mortality rates align with those of the general population, likely due to the younger age and fewer comorbidities of these patients [10]. Mortality rates in IE can range from 6-30%, influenced by factors such as systemic embolic events, which have a mortality rate of 20% compared to 12% for those without. As in our patient, the most common sites for systemic embolization were the central nervous system (48-65%), extremities (30%), spleen (19-32%), and kidneys (6-14%) [11]. Notably, systemic emboli to the spleen and kidneys often co-occur with cerebral emboli. Several risk factors for systemic embolization in IE include vegetation size greater than 10 mm [12], mobile and multiple vegetations, left-sided endocarditis, annular abscess, and Staphylococcus aureus involvement. In this case, the most significant risk factors were anti-coagulation use and a delay in antibiotic therapy [11].

Anti-coagulation was discontinued upon the diagnosis of IE, as it does not reduce the risk of recurrent embolization and can increase the risk of hemorrhagic conversion of strokes [13]. Previously, anti-coagulation was thought to mitigate cerebral embolization by reducing vegetation size; however, in this patient, recurrent embolization persisted despite anti-coagulation and, later, antibiotics. This suggests that anti-coagulation failed to decrease our patients' systemic embolization risk. This case is particularly noteworthy as the patient experienced a stroke during the third week of antibiotic therapy, a period when the risk of systemic embolization is typically lower and despite evidence of healing vegetations on TEE, indicating the need for early surgical evaluation.

Our patient fulfilled the modified Duke's criteria for diagnosing infective endocarditis, exhibiting positive blood cultures, echocardiographic evidence of vegetation, multiple septic embolic events before diagnosis, and a subsequent stroke despite ongoing intravenous antibiotic therapy. According to the European Society of Cardiology guidelines, patients with aortic or mitral valve vegetations larger than 10 mm and experiencing one or more embolic events despite antibiotic treatment should be evaluated for surgical intervention. Other criteria include vegetation >30 mm or vegetation >10 mm and severe native or prosthetic valve disease; the patient is at low operative risk [14]. Initially, our patient did not meet the criteria for surgery. This case underscores the critical importance of proactive surgical evaluation to prevent strokes and systemic embolization, particularly in patients with multiple small vegetations and BAV. Early intervention may be crucial, especially before antibiotic therapy fails to halt embolic events in such patients.

## Conclusions

Our case highlights the challenges in managing infective endocarditis, particularly in young patients with BAV. Early recognition through thorough history-taking and examination is crucial; however, in some cases, vegetation can be difficult to visualize on a TTE, and symptoms may present subacutely. Historically, anti-coagulation was believed to reduce the risk of cerebral embolization by decreasing vegetation size. In this patient, however, recurrent embolization occurred despite anti-coagulation, suggesting that this approach did not effectively reduce the risk of systemic embolization. Therefore, we recommend against using anti-coagulation in cases of infective endocarditis, particularly when it may delay necessary surgical intervention. Additionally, young patients with BAV should be evaluated for early aortic valve replacement, regardless of vegetation size, to help prevent severe complications such as embolic events and strokes.
